# Short and Long-Term Outcomes of Kidney Transplant Recipients Diagnosed With COVID-19 Infection: A Single-Center Observational Study

**DOI:** 10.7759/cureus.31375

**Published:** 2022-11-11

**Authors:** Gina Defelice, Sixto Giusti, Hoonbae Jeon, Mary Killackey, Anil Paramesh, Adarsh Vijay

**Affiliations:** 1 General Surgery, Tulane University School of Medicine, New Orleans, USA; 2 Transplant Nephrology, University of Colorado Anschutz Medical Campus, Aurora, USA; 3 Transplant Surgery, Tulane University School of Medicine, New Orleans, USA

**Keywords:** coronavirus, coronavirus disease 2019, covid-19, acute kidney injury, kidney function, aki outcome, kidney transplant, covid, outcomes

## Abstract

Purpose: Kidney transplant recipients (KTRs) are at an increased risk of severe disease and death caused by coronavirus disease 2019 (COVID-19) infection. There is a paucity of information on the evolution of graft function among hospitalized KTRs who overcome the infection.

Methods: The study included adult KTRs at a single transplant institute who were diagnosed with COVID-19 and needed hospitalization between March 15, 2020, and January 15, 2021. We analyzed patient demographics, comorbid risk factors, and inpatient clinical courses for patients who were able to recover from the infection. Kidney function was analyzed pre-infection, during initial hospitalization, and up to 12 months post-infection.

Results: We identified 48 KTRs who were diagnosed with COVID-19 infection during the study period. Eighteen KTRs among these needed hospitalization for symptoms of fever and respiratory distress. Four patients died of COVID-19 infection-related complications and were excluded from the study. The 14 remaining patients in the study were predominantly of the Black race (85.7%), with a median time since transplant of four years. Of the patients, 64.3% developed acute kidney injury (AKI), with an average peak serum creatinine (sCr) of 2.6 mg/dl and a glomerular filtration rate (GFR) of 35. The mean sCr and GFR of the group were 2 mg/dl and 44 at baseline (prior to infection). This represented an increase in their sCr and GFR of 34% and 29%, respectively. The median follow-up post-infection was 14.5 months. sCr and GFR were 1.87 mg/dl and 47 at three to six months, and 1.89 mg/dl and 48 at nine to 12 months post-infection. New onset proteinuria was noted in five out of 14 patients (36%), with complete resolution of the same in all at three to six months follow-up. Of patients with AKI, 78% had complete recovery at three to six months follow-up. The mean baseline sCr and GFR of patients who had incomplete recovery was 2.35 and 31.5 with pre-existing proteinuria. Of our entire cohort, there was only one patient who experienced graft loss. This patient had a baseline sCr and GFR of 3.8 mg/dl and 22, existing proteinuria on urinalysis, and a history of biopsy-proven rejection.

Conclusion: AKI is common among KTRs who are hospitalized with COVID-19 infection. Most of these recovered, although we noted that patients with baseline lower kidney function and existing proteinuria had a lower recovery rate.

## Introduction

The Municipal Health Commission in Wuhan, China reported its first cases of viral pneumonia that would later be attributed to severe acute respiratory syndrome coronavirus 2 (SARS-CoV-2) in December 2019 [1]. At the time of manuscript preparation, the World Health Organization has reported over 356 million reported cases and 5.6 million deaths attributed to coronavirus disease 2019 (COVID-19) [2]. Kidney transplant recipients (KTRs) are at an increased risk of severe disease and death caused by COVID-19 infection. There is a paucity of information on the evolution of graft function among hospitalized recipients who overcome the infection. This article was previously presented as a meeting abstract at the American Society of Nephrology (ASN) Kidney Week, November 4-7, 2021.

## Materials and methods

This was an institutional review board (IRB)-approved (reference number: 2020-732), single-center, retrospective cohort study of adult KTRs with reverse transcription-polymerase chain reaction (RT-PCR)-confirmed COVID-19 hospitalized at a single transplant center in New Orleans, Louisiana between March 15, 2020, and January 15, 2021. A waiver of informed consent was granted by the institutional IRB. Inclusion criteria included kidney transplant patients with a functioning allograft who were older than 18 years. KTRs who succumbed to the COVID-19 virus were excluded from the study. We performed an electronic medical record chart review to collect and analyze patient demographics, comorbid risk factors, maintenance immunosuppression, patient presentation, immunosuppression modifications, inpatient clinical course, and graft outcomes. Kidney function was analyzed before infection, during initial hospitalization, and up to 12 months following infection. We used serum creatinine (sCr), glomerular filtration rate (GFR), and proteinuria as surrogate markers for kidney function. A standardized form using a Microsoft Excel sheet (Microsoft Corporation, Redmond, WA) was used to extract relevant information. Descriptive statistics were used to examine the cohorts. Categorical values were reported as frequencies, represented as n (%), and examined by chi-square analysis. Continuous variables were reported as median (interquartile range (IQR): 25-75) and examined by the Mann-Whitney U test.

## Results

Cohort demographics and baseline characteristics

During the study period, we identified 48 patients who were diagnosed with RT-PCR-confirmed COVID-19 between March 15, 2020, and January 15, 2021. Of these patients, 18 necessitated hospitalization (37.5%). Four of the patients (22.2%) succumbed to COVID-19 and were excluded from the study. The median patient age was 47 years (IQR: 43.25-51.5), and the majority of patients (71.4%) were male. The overwhelming majority of the patients in the study were of the Black race (85.7%). The median transplant vintage (time after transplant surgery) was nearly four years (IQR: 2.5-14 years). Only one patient was within their first year of transplantation.

Of co-morbidities, hypertension was the most prevalent among our patient cohort (85.7%), followed by obesity (35.7%) and diabetes mellitus (25%). One patient had a history of active or prior malignancy. None of our patients reported a history of underlying asthma or chronic obstructive lung disease (COPD). Four patients (28.6%) received a living donor renal transplant, and the remaining 10 (71.4%) received a deceased donor renal transplant. Most patients (9/14) received the combination of a calcineurin inhibitor (CNI), mycophenolate mofetil or mycophenolic acid (MMF/MPA), and prednisone. Among our cohort, 42.9% had a history of prior opportunistic infection, and 28.6% had a history of allograft rejection. The mean baseline sCr, a measurement taken from laboratory results within three months prior to COVID-19 diagnosis, was 2 ± 0.70 mg/dl. Baseline GFR was 44 ± 15, and six patients had proteinuria on routine monitoring urinalyses. Table 1 details further cohort demographics.

**Table 1:** Cohort demographics All continuous variables are reported as median (IQR: 25-75). Categorical values are frequencies reported as n (%). IQR = interquartile range.

		Number	Percentage
Total patients admitted		14	
Median age (years)		47 (IQR: 43.25-51.5)	
Male		10	71.4%
Race	Black	12	85.7%
	White	2	14.3%
Median transplant vintage (months)		47.5 (IQR: 30-168.25)	
Transplant vintage	<12 months	1	7.1%
	12-24 months	2	14.3%
	>24 months	11	78.6%
Co-morbidities	Hypertension	12	85.7%
	Obesity, BMI > 30	5	35.7%
	Diabetes mellitus	3	21.4%
	History of active or prior malignancy	1	7.1%
Native kidney disease	Hypertension	8	57.1%
	Hypertension and diabetes mellitus	2	14.3%
	Hypertension and human immunodeficiency virus (HIV)	1	7.1%
	Lupus nephropathy	1	7.1%
	Polycystic kidney disease	1	7.1%
	Neurogenic bladder	1	7.1%
Chronic kidney disease (CKD) staging	Stage 1	0	0%
	Stage 2	2	14.3%
	Stage 3	5	35.7%
	Stage 4	3	21.4%
	Stage 5	4	28.6%
Renal donor type	Deceased	10	71.4%
	Living	4	28.6%
Maintenance immunosuppression	Calcineurin Inhibitor (CNI)	14	100%
	Mycophenolic acid (MPA)	11	78.6%
	Prednisone	12	85.7%
History of opportunistic infection		6	42.9%
History of rejection		4	28.6%

Hospital course and clinical outcomes

Admission data and clinical outcomes are presented in Table 2. Of the 14 patients admitted who survived, the median duration of symptoms prior to admission was 6.5 days (IQR: 4.25-7). The most prevalent symptoms on presentation were cough (42.9%) and fever (35.7%), followed by dyspnea (21.4%). Of the patients, 21.4% reported nausea or vomiting, myalgia, and fatigue on presentation, and 14.3% reported diarrhea. Patients were hospitalized for a median of three days (IQR: 2-6). The most common immunosuppression regimen adjustment was to change or withdraw MMF/MPA (64.3%), whereas only 28.6% had their CNI dose adjusted or withdrawn.

**Table 2:** Presentation and clinical outcomes All continuous variables are reported as median (IQR: 25-75). Categorical values are frequencies reported as n (%). IQR = interquartile range.

		Number	Percentage
Total patients admitted		14	
Duration of symptoms prior to admission (days)		6.5 (IQR: 4.25-7)	
Presenting symptoms	Fever	5	35.7%
	Cough	6	42.9%
	Nausea/vomiting	3	21.4%
	Myalgia	3	21.4%
	Diarrhea	2	14.3%
	Dyspnea	3	21.4%
	Fatigue	3	21.4%
Length of hospitalization (days)		3 (IQR: 2-6)	
Immunosuppressant modulation	Held/adjusted calcineurin inhibitor (CNI)	4	28.6%
	Held/adjusted mycophenolic acid (MPA)	9	64.3%
	Held/adjusted steroids	3	21.4%
Outcomes	Urine analysis (U/A) with proteinuria	11	78.6%
	Nasal cannula	2	14.3%
	Mechanical ventilation	0	0%
	Acute kidney injury (AKI)	9	64.3%
	Required in-patient dialysis	1	7.1%
	Graft loss	1	7.1%

On admission, COVID-19-specific acute phase reactants were measured. Average C-reactive protein (CRP), ferritin, procalcitonin, lactate dehydrogenase (LDH), and D-dimer were reported at 6.6 mg/L, 1716.5 ng/mL, 0.45 ng/mL, 277 U/L, and 3.4, respectively (Table 3).

**Table 3:** Average laboratory values prior to, during, and following hospitalization SD = standard deviation; mg/L = milligrams per liter; ng/mL = nanograms per milliliter; U/L = units per liter; k/ul = thousands per cubic milliliter; gm/dl = grams per deciliter; FEU = fibrinogen equivalent units.

	Admission	3-6 months follow-up	9-12 months follow-up
Number of patients	14	14	11
C-reactive protein (CRP) (mean ± SD, mg/L)	6.6 ± 9.8		
Ferritin (mean ± SD, ng/mL)	1716.5 ± 1156.9		
Procalcitonin (mean ± SD, ng/mL)	0.45 ± 0.44		
Lactate dehydrogenase (LDH) (mean ± SD, U/L)	277 ± 82		
White blood count (WBC) (mean ± SD, k/ul)	6.4 ± 2.5	7.06 ±1.92	7.24 ± 1.88
Hemoglobin (mean ± SD, gm/dl)	12.0 ± 2.8	11.2 ± 1.56	12.2 ± 1.94
Platelet (mean ± SD, k/ul)	192 ± 90	238 ± 96	257 ± 87
D-dimer (mean ± SD, mg/L FEU)	3.4 ± 5.8		

During hospitalization, 11 patients had a urinalysis demonstrating proteinuria. Nine patients (64%) developed acute kidney injury (AKI), defined by KDIGO (Kidney Disease: Improving Global Outcomes) guidelines, with an average peak sCr of 2.6 and a GFR of 34. A single case of graft loss occurred in a patient with a history of biopsy-proven rejection and was resumed on hemodialysis. Most patients were able to remain on room air, but two required a nasal cannula. Case rate mortality was 22.2% (four of 18 total hospitalized patients); three of these four patients were recorded to have experienced an AKI during hospitalization and we lack data for the remaining patient.

Post-discharge follow-up

The median follow-up post-COVID-19 infection was 14.5 months (IQR: 10.4-16.7 months). The recipients had a mean sCr and GFR of 1.87 mg/dL and 47 at six months follow-up and 1.88 mg/dl and 48 at 12 months follow-up, respectively. All five cases of new-onset proteinuria were completely resolved within three months of follow-up (Table 4).

**Table 4:** Kidney function prior to, during, and following hospitalization SD = standard deviation; mg/L = milligrams per liter.

	Baseline	Admission	3-6 months follow-up	9-12 months follow-up
Serum creatinine (mean ± SD, mg/dL)	2 ± 0.7	2.6 ± 1.3	1.87 ± 0.51	1.89 ± 0.67
Glomerular filtration rate (GFR) (mean ± SD)	44 ± 15	35 ± 15	47 ± 18	48 ± 19
Patients with proteinuria	6	11	6	6

All but two patients (78%) with AKI during hospitalization had complete recovery by six-month follow-up. These two recipients continue to experience compromised kidney function at nine months, with sCr values of 2.8 and 3.2 mg/dL from baselines of 2 and 2.7 mg/dL, respectively. The mean baseline sCr and GFR of patients who had incomplete recovery were 2.35 mg/dL and 31.5, respectively. This is in comparison to the overall baseline average sCr and GFR of patients (2 mg/dL and 44).

Finally, all patients had returned to their original maintenance immunosuppression by three months post-discharge.

## Discussion

The burden of COVID-19 on kidney transplant recipients

In the general population, the case mortality ratio is estimated to be around 2% at the time of manuscript preparation [2]. However, in the KTR population, multi-center cohort studies have reported a case mortality ratio of 14-32% [3-12]. Studies in the general population have reported 0.5-7% of patients developing an AKI [1,13,14], in comparison to 15.3-52% of KTRs [3-12]. The data from our cohort correspond with values reported by other similarly sized cohort studies and multi-center cohorts of kidney transplant patients.

The pathophysiology of COVID-19-related AKI has been reasoned to be multifactorial with local immune cell infiltration having a key role in addition to microvascular thrombi and intravascular coagulation in the affected kidney [15]. Though there is more evidence currently toward reducing the severity of COVID-19-related AKI in transplant recipients with the use of steroids and interleukin 6 (IL-6) receptor antagonists, treatment for patients included in our study was guided by institutional protocols based on the evidence available at the onset of the COVID-19 pandemic and kept evolving with time [16]. These protocols included stopping the use of mycophenolate and individual clinical judgments on the use of remdesivir, low-dose methylprednisolone, and colchicine.

Recovery of kidney function following AKI

Importantly, our data reveal that the majority of those who survived hospitalization with COVID-19 recover their kidney function. Of the nine patients who had developed an AKI and recovered from COVID-19, seven returned to baseline sCr within six months of follow-up. The two remaining patients with incomplete recovery of their graft function had lower baseline kidney function, with an average baseline sCr and GFR of 2.35 and 31.5 with pre-existing proteinuria. Of our entire cohort, there was only one patient who experienced graft loss. This patient had a baseline sCr of 3.8 mg/dL, baseline GFR of 22, existing proteinuria on urinalysis, and a history of biopsy-proven rejection.

Notably, recovery of kidney function has been shown to occur relatively rapidly, with 61% of a Swiss cohort experiencing in-hospital renal recovery at discharge and 40.5% of a large multi-center cohort experiencing complete recovery before three months follow-up [17,18]. Our smaller study corroborates these data, as 66.6% of our patients had graft recovery by three months. However, of those who do not recover, it appears that graft function was less optimal at baseline. Bajpai and colleagues found that patients who did not recover from their AKIs had lower kidney function and were more likely to have lower estimated GFRs and proteinuria at baseline, a history of graft rejection, and orthostatic hypotension [18]. Baseline proteinuria and orthostatic hypotension independently predicted incomplete graft recovery in their study. Thus, patients with existing graft dysfunction may warrant special precaution as the circulation of the virus continues.

## Conclusions

The descriptive nature of our relatively small retrospective cohort study does not enable us to make any statements on causation. Furthermore, we must consider that we lack results from the era of unavailable-to-limited testing during the beginning of the pandemic. It is thus difficult to draw larger conclusions on patient outcomes, as many patients who died may not have been tested, and only patients who were capable to go to the hospital and being tested were included. While patient treatment regimens were guided by institutional protocol, management was subject to individual clinician judgment and rapidly evolved over the course of the pandemic. Outcomes due to changes in immunosuppression regimens are largely unstudied and warrant investigation.

In our study, acute kidney injury was common among KTRs who were hospitalized with COVID-19 infection. Our data reveal that the majority of those who survive hospitalization with COVID-19 recover their kidney function. In our cohort, patients with baseline lower kidney function and existing proteinuria had a lower recovery rate. The authors look forward to the continued improvement of COVID-19 management and a return to normalcy.
